# Usefulness of the Long Cap-Assisted Method for Securing Countertraction During Endoscopic Submucosal Dissection

**DOI:** 10.14309/crj.0000000000001807

**Published:** 2025-08-18

**Authors:** Nobutaka Doba, Kosuke Shibayama, Shinzo Abe, Daiki Sakuma, Masanobu Someya, Kazuto Komatsu, Shin Maeda

**Affiliations:** 1 Department of Gastroenterology, Yokosuka City Hospital, Nagasaka, Yokosuka, Japan; 2 Department of Gastroenterology, Yokohama City University Graduate School of Medicine, Yokohama, Japan

**Keywords:** cap-assisted endoscopy, endoscopic submucosal dissection, countertraction, long cap

## Abstract

Endoscopic submucosal dissection (ESD) is effective but technically challenging, often requiring traction devices that involve multiple clips and added time. The long cap-assisted method, using a transparent cap with an extended tip, offers a simple alternative. In 2 cases—a 65-year-old with early gastric cancer and an 81-year-old with a large colonic laterally spreading tumor—standard caps failed to provide sufficient countertraction. Switching to a long cylindrical cap (not tapered) improved submucosal visualization and facilitated dissection. To our knowledge, this is the first report to specifically describe the long cap's use in achieving countertraction during endoscopic submucosal dissection.

## INTRODUCTION

Endoscopic submucosal dissection (ESD) enables en bloc resection of gastrointestinal lesions but is more technically demanding than endoscopic mucosal resection (EMR). Successful ESD requires a stable submucosal view and adequate tension on the dissection plane.^1^ Although caps or changes in body position using gravity are commonly employed to provide tension, these methods often fall short. Toward the end of the procedure, the specimen becomes mobile, reducing countertraction, slowing dissection, and prolonging procedure time.

Although various traction devices have been developed, most require multiple clips and increase procedural complexity.^2–6^ We have effectively used a long cap-assisted method—a transparent cylindrical (not tapered) cap with an extended tip—to achieve countertraction, especially in the later stages of ESD. We present 2 cases (gastric and colorectal) illustrating the use of this method.

## CASE REPORT

### Case 1

**Figure 1.**
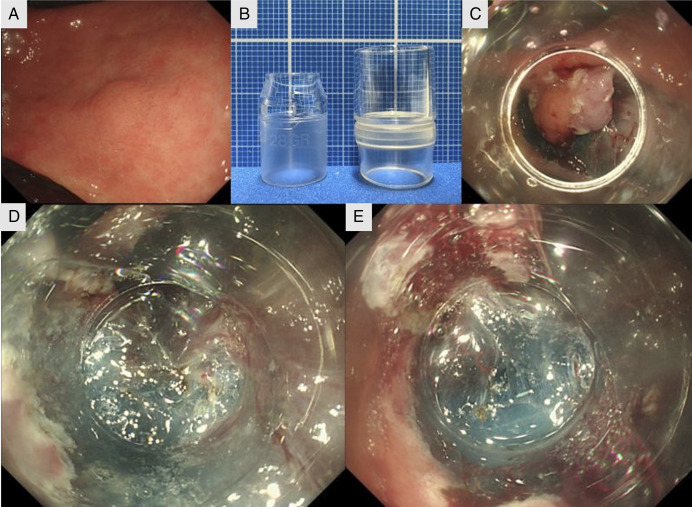
(A) Pretreatment endoscopic image showing a 5 mm 0-IIa lesion in the lesser curvature of the gastric angulus. (B) Left: Standard cap (DH-28 GR; tip opening, 8 mm; protrusion length, 7 mm; Fujifilm). Right: Long cylindrical cap (MH-463; diameter, 13.5 mm; protrusion length, 12 mm; Olympus). (C) Endoscopic image before cap replacement showing insufficient countertraction at the end of the dissection. (D) Endoscopic image after cap replacement demonstrating improved countertraction and clear submucosal visualization. (E) Endoscopic image after resuming dissection, with a clearly visible and easily defined dissection line.

A 65-year-old man was referred for treatment of a 5 mm early gastric cancer located on the lesser curvature of the angulus (Figure 1). ESD was performed using a GIF-H290ZI (Olympus, Tokyo, Japan) endoscope with a standard cap (DH-28 GR, tip opening 8 mm, protrusion 7 mm, Fujifilm, Tokyo, Japan) (Figure 1). Initially, dissection proceeded smoothly with good submucosal visibility, but countertraction weakened toward the end (Figure 1). Although the procedure could be completed, a traction device might have made it faster and more reliable. Owing to concerns about cost and time, we chose not to use one.

Instead, we applied a long cap-assisted method, expecting the longer tip to lift the specimen and improve countertraction. The endoscope was withdrawn, and the cap was replaced with a long cylindrical cap (MH-463, 13.5 mm diameter, 12 mm protrusion, Olympus) (Figure 1). After reinsertion, the cap was placed beneath the specimen, providing good countertraction and clear visibility of both submucosal edges (Figure 1). The scope exchange took 94 s, after which dissection resumed smoothly with a clear submucosal view (Figure 1).

The procedure was performed using the TriangleTipKnifeJ (4 mm tip protrusion, Olympus) and the ITknife2 (4 mm tip protrusion, Olympus), following a classic endoscopic submucosal dissection (ESD) technique. During the long cap-assisted method, submucosal dissection was primarily performed using the ITknife2 (Olympus).

### Case 2

**Figure 2.**
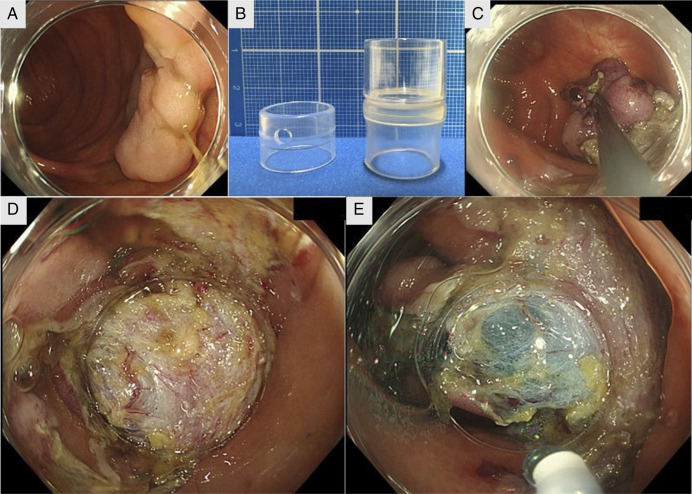
(A) Pretreatment endoscopic image showing a 4 cm 0-IIa lesion in the ascending colon. (B) Left: Standard cap (D-201-12704; diameter, 13.4 mm; protrusion length, 4 mm; Olympus). Right: Long cylindrical cap (MH-466; diameter, 17.2 mm; protrusion length, 12 mm; Olympus). (C) Endoscopic image before cap replacement showing poor countertraction and difficulty identifying the dissection line. (D) Endoscopic image after cap replacement showing improved countertraction, clear visualization of both submucosal edges, and a well-defined dissection line. (E) Endoscopic image after resuming the procedure, showing successful resection with sustained submucosal visibility.

An 81-year-old man was referred for treatment of a 4 cm laterally spreading tumor in the ascending colon (Figure 2). ESD was performed using a PCF-260AZI endoscope (Olympus) with a standard cap (D-201-12704, 13.4 mm diameter, 4 mm protrusion) (Figure 2). As dissection progressed, countertraction weakened, making it difficult to set the dissection line (Figure 2).

No traction device was available, so based on previous gastric ESD experience, we applied the long cap-assisted method. The endoscope was removed and replaced with a CF-HQ290ZI (Olympus), and the cap was switched to a long cylindrical type (MH-466, 17.2 mm diameter, 12 mm protrusion) (Figure 2). After reinsertion, the cap was positioned beneath the specimen, significantly improving the field of view and clearly exposing both submucosal edges (Figure 2). The total time to resume treatment was 9 min (3 min for scope and cap exchange, 6 min to reach the cecum). Subsequently, the dissection line was easily identified, and the procedure was completed smoothly with continuous visualization of both edges (Figure 2).

The procedure was performed using the HookKnifeJ (4 mm tip protrusion, Olympus) and the ITknife nano (3.5 mm tip protrusion, Olympus), following a classic ESD technique. During the long cap-assisted method, submucosal dissection was primarily conducted using the ITknife nano (Olympus).

## DISCUSSION

Adequate countertraction is essential for smooth performance of ESD.^1^ However, as dissection progresses, the specimen becomes increasingly mobile, making countertraction more difficult. Insufficient countertraction during the final phase often prolongs the procedure.

To address this, various traction devices have been developed,^2–6^ which can be classified into 3 main types. The clip-with-line method uses dental floss or silk thread tied to a clip attached to the specimen, with the thread exiting the body.^2,3^ This method is simple and low-cost but may interfere with the endoscope and lacks directional control. The internal traction method involves attaching a clip with a spring or rubber ring to the specimen and anchoring it to the opposite lumen wall.^4,5^ This allows directional control without interfering with the endoscope, but at a higher cost. The external traction method applies traction externally using forceps or a snare,^6^ allowing directional adjustment but also increasing interference and cost. Owing to these cost and time concerns, operators may hesitate to use these devices, especially late in the procedure.

The long cap-assisted method has demonstrated use in various endoscopic procedures. Long caps, with extended tip protrusion, provide a wider field of view and increased working space compared with standard caps. In esophageal food bolus obstruction, this technique improves en bloc removal rates and shortens procedure time.^7^ It also reduces closure time in endoscopic closure of gastrointestinal defects^8,9^ and increases en bloc resection rates for 20–30 mm lesions in colorectal EMR.^10^ In difficult stenting cases for GI stenosis, it facilitates easier stent placement.^11^

In ESD, the long cap-assisted method provides effective countertraction by elevating the specimen with the cap tip. Traction direction can be adjusted through scope manipulation, and the technique avoids interference with the endoscope. As it requires no clips and uses reusable caps, it is also cost-effective (Table 1). However, because it requires space beneath the specimen, its application is limited during the early phase of dissection.

**Table 1.** Comparison of various traction methods and the long cap-assisted method in endoscopic submucosal dissection

	Clip-with-line method	Internal traction method	External traction method	Long cap-assisted method
Clip	Necessary	Necessary	Necessary in many cases	Unnecessary
Traction direction	Unadjustable	Adjustable	Adjustable	Adjustable
Interference with endoscope	Existence	None	Existence	None

In Case 1, although the procedure could have been completed without cap replacement, the long cap effectively secured the submucosal field (Figure 2). The method involved simply replacing the cap and reinserting the scope, requiring only 94 s. In Case 2, the dissection line became unclear mid-procedure, and no traction device was available. The long cap-assisted method resolved the issue, allowing the procedure to proceed smoothly with consistent submucosal visibility (Figure 2).

A PubMed search using the terms “long hood” and “ESD” revealed only one report—a pharyngeal ESD case that used a long transparent cap (Elastic Touch, Top) to create working space by pushing down the larynx.^12^ However, that report focused on space creation rather than traction. To our knowledge, no previous report has specifically described the use of the long cap-assisted method in achieving countertraction during ESD.

The long cap-assisted method facilitates countertraction by elevating the specimen with the tip of the cap, enabling the operator to modulate the direction of traction through scope angulation. Although this countertraction effect may lift the mucosa, no adverse events such as muscle layer injury were observed in our cases. This technique is compatible with a variety of endoscopic knives, including both insulated and noninsulated types, and is applicable across multiple ESD approaches, including the classic, tunneling, and pocket methods. However, compared with standard caps, this method necessitates a greater extension of the knife beyond the endoscope tip. Consequently, knives with a short tip protrusion may have their tips obscured within the cap, potentially impairing visibility. For this reason, we prefer to use knives with a longer tip protrusion length. In addition, we acknowledge that this technique may be less suitable for underwater ESD due to the potential accumulation of fluid and air bubbles within the cap, which can compromise the endoscopic field of view.

The long cap lacks a side hole, and some fluid accumulation was noted, though it did not significantly impair visibility. In our experience, the cap did not interfere with hemostasis or the application of through-the-scope (TTS) clips. On the contrary, it proved advantageous by providing sufficient working space and allowing for greater maneuverability of the cap. Compression hemostasis using the protruding tip of the long cap, along with the prevention of blood inflow into the cap, is useful for maintaining a clear endoscopic field during hemostasis. In addition, the wide inner lumen of the cap allows for the smooth insertion and removal of devices such as hemostatic forceps and TTS clip applicators without compromising visualization. We have also frequently experienced successful retrieval of blood clots or specimens by aspirating them into the cap and withdrawing the endoscope, without the need for additional tools such as forceps or retrieval nets.

Although scope exchange may add time, particularly in the right colon, improved visibility and procedural stability can outweigh this disadvantage. Cost-effectiveness depends on whether reusable caps are available; in some settings, the cost may be comparable to the clip-with-line method.

These observations suggest that the long cap-assisted method is a simple, low-cost, and effective alternative to conventional traction techniques, especially when sufficient space exists beneath the specimen.

## DISCLOSURES

Author contributions: N. Doba: Conceptualization, writing—original draft preparation, writing—review and editing, visualization; K. Shibayama: data curation; S. Abe and D. Sakuma: investigation; M. Somey: data curation; K. Komastu and S. Maeda: supervision.

Financial disclosure: None to report.

Informed consent was obtained for this case report.

## ABBREVIATIONS:

ESD: endoscopic submucosal dissection
EMR: endoscopic mucosal resection
TTS: through-the-scope
